# 

**Published:** 1885-05

**Authors:** 


					﻿
MAY, 1885.

OLD SERIES
Vol XXV.

;XiEM?serie£
[, N° ³ /

fcANTft

•w i ■ nr

■ ■ I ■ ■             /fu ' ■

(Journal

!> W.F. WESTMORELAND. M
H.V.M.MILLER.M.D.LL.D.J
JAMES.A.GRAY. M.D

JAMES.P.HARRISON'
  Atlanta,ga.

SUBSCRIPTION : $2.50 A YEAR.
				

